# Transcription Factor Retinoid-Related Orphan Receptor γt: A Promising Target for the Treatment of Psoriasis

**DOI:** 10.3389/fimmu.2018.01210

**Published:** 2018-05-30

**Authors:** Lipeng Tang, Xiaozhi Yang, Yongxin Liang, Hesong Xie, Zhenhua Dai, Guangjuan Zheng

**Affiliations:** ^1^Department of Pharmacology of Traditional Chinese Medicine, The Second Affiliated Hospital of Guangzhou University of Chinese Medicine, Guangzhou, Guangdong, China; ^2^School of Bioscience and Bio-Pharmaceutics, Guangdong Pharmaceutical University, Guangzhou, Guangdong, China; ^3^Section of Immunology, The Second Affiliated Hospital of Guangzhou University of Chinese Medicine, Guangzhou, Guangdong, China; ^4^Department of Pathology, The Second Affiliated Hospital of Guangzhou University of Chinese Medicine, Guangzhou, Guangdong, China

**Keywords:** autoimmune disorder, psoriasis, T helper 17 cells, retinoid-related orphan receptor γt nuclear receptor, retinoid-related orphan receptor γt inverse agonist

## Abstract

Psoriasis, which is a common chronic inflammatory skin disease, endangers human health and brings about a major economic burden worldwide. To date, treatments for psoriasis remain unsatisfied because of their clinical limitations and various side effects. Thus, developing a safer and more effective therapy for psoriasis is compelling. Previous studies have explicitly shown that psoriasis is an autoimmune disease that is predominantly mediated by T helper 17 (Th17) cells, which express high levels of interleukin-17 (IL-17) in response to interleukin-23 (IL-23). The discovery of the IL-23–Th17–IL-17 axis in the development of psoriasis has led to the paradigm shift of understanding pathogenesis of psoriasis. Although anti-IL-17 antibodies show marked clinical efficacy in treating psoriasis, compared with antibodies targeting IL-17A or IL-17R alone, targeting Th17 cells themselves may have a maximal benefit by affecting multiple proinflammatory cytokines, including IL-17A, IL-17F, IL-22, and granulocyte-macrophage colony-stimulating factor, which likely act synergistically to drive skin inflammation in psoriasis. In this review, we mainly focus on the critical role of Th17 cells in the pathogenesis of psoriasis. Especially, we explore the small molecules that target retinoid-related orphan receptor γt (RORγt), a vital transcription factor for Th17 cells. Given that RORγt is the lineage-defining transcription factor for Th17 cell differentiation, targeting RORγt *via* small molecular inverse agonists may be a promising strategy for the treatment of Th17-mediated psoriasis.

## Introduction

Psoriasis is an autoimmune disease with chronic skin inflammation (1), affecting over 125 million people worldwide (up to 2–4% of the world’s population) (2). It is predominantly a skin disease, which can manifest itself as various phenotypes, including plaque-type psoriasis or psoriasis vulgaris, guttate psoriasis, pustular psoriasis such as palmoplantar pustulosis, and erythrodermic psoriasis.

Psoriasis vulgaris, a most common type of psoriasis, is characterized by well-defined areas of erythematous and plaques with overlying silvery scale. The main histopathological changes of psoriasis vulgaris include abnormal cell proliferation, parakeratosis, hyperkeratosis, angiogenesis, and inflammatory cell infiltration (1, 3).

Increasing evidence has shown that comorbid cardiovascular diseases are the leading causes of death among patients with psoriasis (4). In addition, a high prevalence of metabolic syndrome, psychosocial distress or psychiatric disorders, chronic kidney disease, and gastrointestinal disease has been demonstrated in individuals with psoriasis (5, 6). The global financial burden associated with the care of psoriatic patients is substantial and significant (7–10). It was reported that the annual costs for treating psoriasis in USA amounted to approximately $112 billion in 2013 (11). As for individuals, patients with psoriasis would incur a lifetime medical expense for relief of physical symptoms and emotional health (12).

## Therapeutic Challenges for Psoriasis

Based on the immunological characteristics of psoriasis, researchers have developed topical treatments, including corticosteroids, vitamin D3 analogs and Victoria A acid, and systemic therapies, including methotrexate and cyclosporine, for psoriasis. In clinic, patients with mild-to-moderate plaque psoriasis are usually treated topically with corticosteroids and vitamin D3 analogs, whereas those with moderate-to-severe psoriasis are systemically treated with methotrexate and cyclosporine (13, 14). However, these treatments exhibit low efficacies, poor tolerability, and various adverse reactions (15) (Table 1).

**Table 1:** Traditional treatment for psoriasis.

Traditional treatments	Molecular mechanisms	Adverse reactions
Corticosteroids	Vascular permeability ↓ Skin edema ↓ Neutrophil infiltration ↓ Cell proliferation ↓	Skin atrophy, hair thinning, hypopigmentation, allergic contact dermatitis

Vitamin D3 analogs	Immune modulation Keratinocyte maturation ↓	Hypercalcemia, urinary calcium concentrations increased, tissue calcification

Victoria A acid	The activity of Th1 and Th17 cells ↓ Keratinocyte differentiation	External medicine:itching and burning sensation and erythema, friction at the erythema Oral administration:dry and exfoliated skin, diffuse baldness, denaturation, and skin adhesion

Methotrexate	Inhibition of the enzyme 5-aminoimidazole-4-carboxamide ribonucleotide transformylase Adenosine ↓ Tumor necrosis factor (TNF) and two nuclear factor-κB subunits ↓	Bone marrow toxicity, cirrhosis, nausea, and macrocytic anemia

Cyclosporine	T cell activity ↓	Nephrotoxicity, numerous drug–drug interactions; hypertension, hyperkalemia, increased risk of lymphoma, and squamous cell carcinoma with long-term use

Fumarates	TNF, IL-12, and interleukin-23 production ↓	Gastrointestinal disturbances, flushing, eosinophilia, and proteinuria

Although the introduction of biological treatments, including tumor necrosis factor (TNF)-α antagonists (Efalizumab), anti-TNF antibody (Adalimumab) (16), IL-12/interleukin-23 (IL-23) antagonists (Ustekinumab) (17), and interleukin-17 (IL-17) antagonists (Secukinumab, Ixekizumab, and Brodalumab) (18, 19), has revolutionized the short-term treatment of moderate-to-severe plaque psoriasis, the long-term use of biological therapies may cause loss of efficacy as well as severe adverse reactions, such as infection, cancer, and hepatic dysfunction (20, 21) (Table 2). These clinical side effects of existing treatments strongly suggest that it is still urgent to discover safer and more effective therapeutic drugs for psoriasis.

**Table 2:** Biologic therapies for psoriasis.

Biologic therapies	Molecular targets	Adverse reactions
Efalizumab	Tumor necrosis factor (TNF) receptor fusion protein antagonist	Infections, certain malignancies, particularly cutaneous squamous cell carcinoma

Adalimumab	Anti-TNF human monoclonal antibody	Infections and certain malignancies, particularly cutaneous squamous cell carcinoma Serious adverse reactions:active tuberculosis, myocardial infarction, optic neuritis, pancytopenia, lymphoma, etc.

Ustekinumab	Anti-IL-12 and anti-interleukin-23 human monoclonal antibody	Nasopharyngitis, upper respiratory tract infection, headache, diarrhea, muscle pain, dizziness, etc.

Secukinumab	Anti-IL17A human monoclonal antibody	The development of *Candida* infections Special adverse reaction:neutropenia

Ixekizumab	Anti-IL-17A human monoclonal antibody	The development of *Candida* infections

Brodalumab	Anti-IL-17A receptor human monoclonal antibody	The development of *Candida* infections suicidal ideation

## Pathogenesis of Autoimmune Psoriasis

To develop a better, safer, and more effective therapy for psoriasis, it is imperative to understand psoriatic pathogenesis. Previous studies have indicated that psoriasis is a skin disease mainly mediated by dendritic cells and T cells although macrophages, neutrophilic granulocytes, keratinocytes, vascular endothelial cells, and the cutaneous nervous system are involved in its pathogenesis (22, 23).

**Figure 1:**
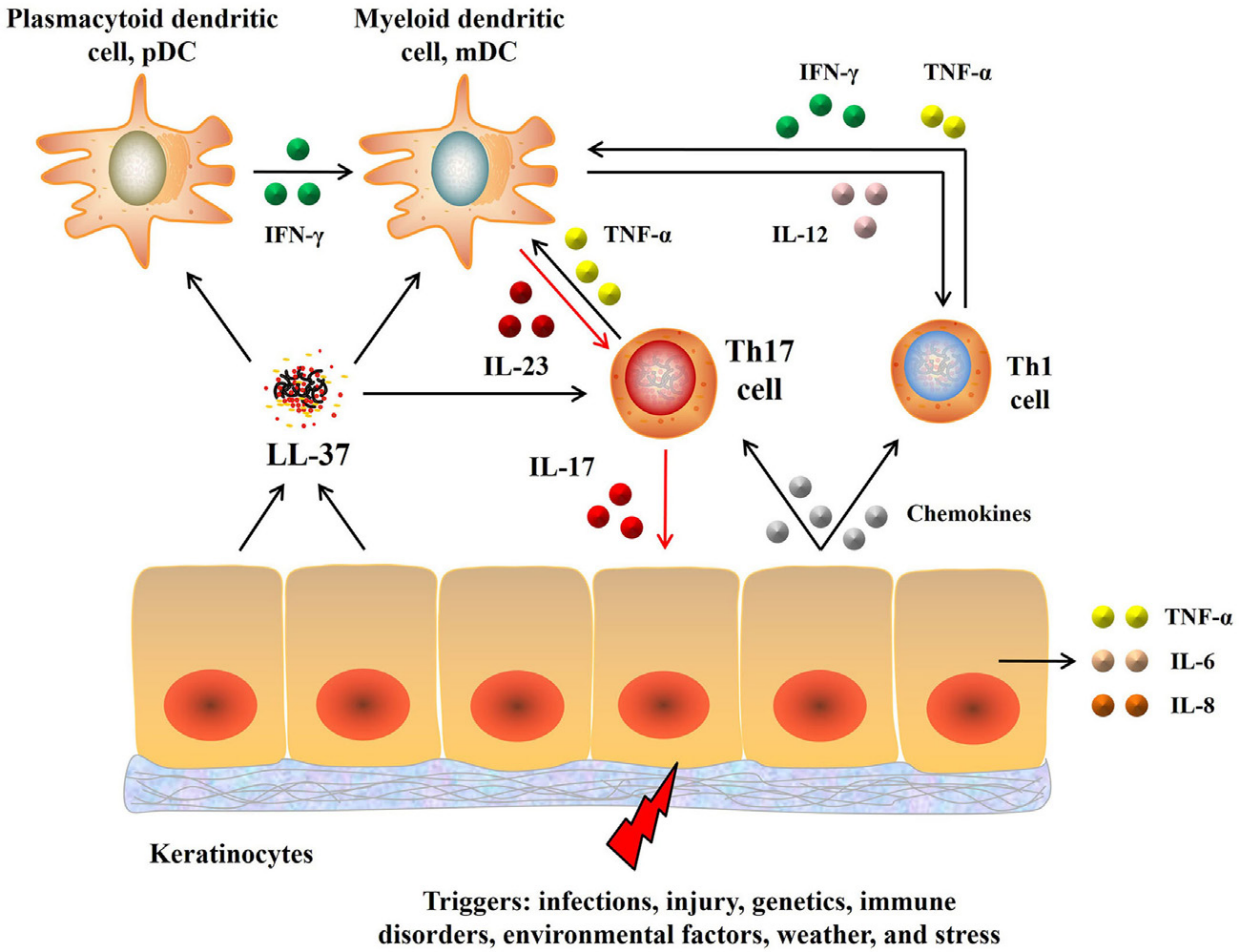
Pathogenesis of psoriasis. Upon activation, keratinocytes secrete LL-37 that in turn activates dendritic cells, which then produce IL-23 and IL-12. IL-23 induces differentiation of naive T cells into Th17 cells that then overproduce IL-17 and IL-22. IL-17 activates keratinocytes, promotes epidermal hyperplasia and recruits proinflammatory cells, resulting in a positive proinflammatory feedback that accelerates the development of psoriasis. Moreover, IL-12 produced by dendritic cells also promotes the differentiation of Th1 cells that in turn produce Th1 cytokines, including IFN-γ. Abbreviations: IL, interleukin; TNF, tumor necrosis factor; IFN-γ, interferon-γ; Th17, T helper 17; IL-23, interleukin-23; IL-17, interleukin-17.

Epidermis-produced antimicrobial peptide LL-37 (cathelicidin), which acts as a dendritic cell activator, is upregulated in the initial phase of psoriasis (24). LL-37 stimulates dermal plasmacytoid dendritic cells to produce interferon-γ (IFN-γ), which in turn activates myeloid dendritic cells (mDCs) to secrete IL-12 and IL-23. IL-12 promotes the differentiation of Th1 cells, whereas IL-23 enhances T helper 17 (Th17) cell development. Th1 cells secrete more IFN-γ and TNF-α to further stimulate mDCs. In addition, Th17 cells secrete IL-17 to stimulate keratinocytes to over-proliferate, causing psoriasis-like lesions (25). Furthermore, the lesion cells secrete a series of chemokines, attracting more immune cells to inflamed tissue, while the damaged cells are digested by macrophages and produce LL-37, forming a positive feedback path that accelerates the development of psoriasis. Recently, LL-37 has been proved to be a T-cell-reactive autoantigen in psoriasis. LL37-specific CD4^+^ T cells can produce Th17-related cytokines (26). In summary, these results indicate that psoriasis is an autoimmune disease mediated by dendritic cells and T-cells (Figure 1).

## The Main Role of Pathogenic Th17 Cells in Psoriasis

T helper 17 cells are a distinct subset of T helper cells that mainly produce IL-17A, IL-17F, and IL-22. Mounting evidence shows that there are two subsets of Th17 lineages. A non-pathogenic subset of Th17 cells induced by TGF-β1 and IL-6 has an important role in host defense against specific pathogens by producing IL-17 and IL-10 (27). The production of IL-10 by non-pathogenic Th17 cells restrains Th17 cell-mediated pathology so that they are incapable of promoting autoimmune inflammation. On the other hand, differentiation of highly pathogenic Th17 cells from naïve T cells occurs in the presence of IL-23, IL-6, and TGF-β1 (28, 29). More precisely, exposure to IL-23 diminishes the anti-inflammatory cytokine IL-10 in developing Th17 cells (27). In addition, IL-23 stabilizes and reinforces Th17 phenotypes by increasing expression of IL-23 receptor (30, 31) and endowing Th17 cells with pathogenic effector functions (32–34). These pathogenic Th17 cells contribute to various autoimmune diseases (35, 36).

Psoriasis is primarily characterized as a Th1-driven disease because the levels of Th1 cytokines, such as IFN-γ, TNF-α, and interleukin (IL)-12, are markedly elevated in psoriatic lesions, while there is no such an increase in expression of Th2 cytokines (IL-4, IL-5, and IL-13) (37–39). With the characterization of a distinct subset of Th17 cells, the research field of psoriasis has experienced a major paradigm shift.

Indeed, previous results have confirmed that pathogenic Th17 cells play a central role in the development of psoriasis(40, 41). Pathological or immunohistochemical studies on psoriasis have shown that skin lesions are mainly infiltrated by Th17 cells. In addition, IL-23, which is produced by activated mDCs, drives naïve T cells to develop into pathogenic Th17 cells (42). IL-17, which is predominantly produced by pathogenic Th17 (43), is significantly elevated in patients with psoriasis compared with healthy subjects. Upregulated IL-17 has potent ability to recruit neutrophils (44, 45), to activate T cells, to stimulate fibroblasts (46), and to promote development of multiple lineages of macrophages (47, 48). Moreover, pathogenic Th17-secreted IL-17 induces proliferation of keratinocytes and secretion of antimicrobial peptides, cytokines, and chemokines, which in turn recruit more immune cells to inflamed tissue. This positive feedback loop between Th17 cells and keratinocytes has been proved to contribute to the chronic inflammatory phase of psoriasis (43, 49, 50). Other proinflammatory factors released by pathogenic Th17 cells, such as IL-22, TNF-α, and granulocyte-macrophage colony-stimulating factor (GM-CSF), stimulate keratinocytes to release chemokines, further sustaining the inflammatory cycle to promote the development of psoriasis (51, 52).

## Retinoid-Related Orphan Receptor γt (RORγt): A Lineage-Defining Transcription Factor for Th17 Cells

**Figure 2:**
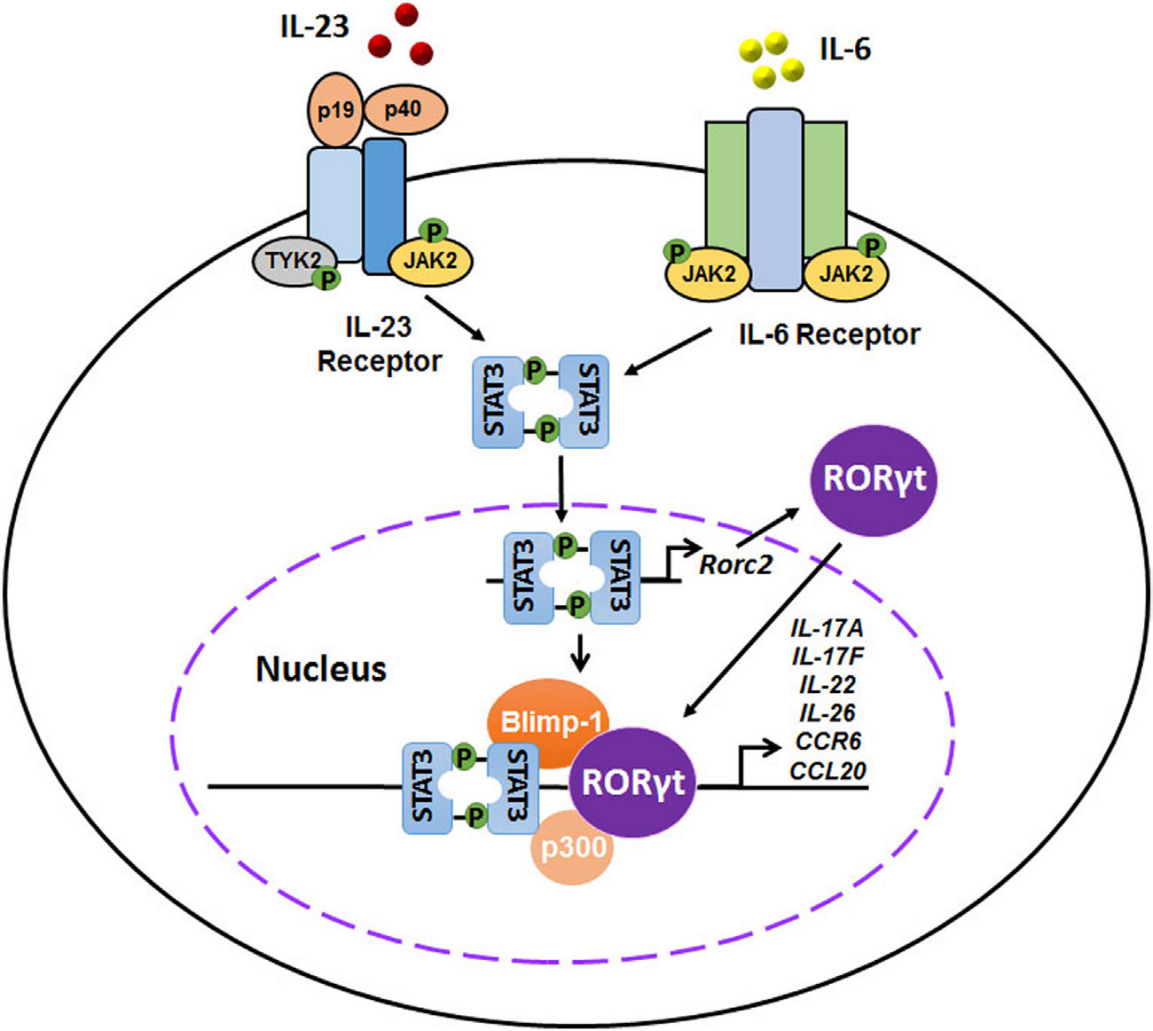
Interplays of interleukin-23 (IL-23), IL-6, signal transducer and activator of transcription 3 (STAT3), and retinoid-related orphan receptor γt (RORγt) in the differentiation of pathogenic T helper 17 (Th17) cells. IL-23 and IL-6 signals activate the JAK–STAT signaling pathway, inducing a strong phosphorylation and dimerization of STAT3. STAT3 homodimers induce the expression and nuclear translocation of RORγt, which in turn promotes Th17 responses by activating Th17 gene promoters, including *Il17a, Il17f, Il22, Il26, Il23r, Csf-2, Ccr6*, and *Ccl20*. In addition, IL-23 signaling-induced transcription factor Blimp-1 enhances pathogenic Th17 function by co-localizes RORγt and STAT-3 at *Il17a, Il23r*, and *Csf-2* enhancer sites.

The differentiation of Th17 cells, similar to that of Th1 and Th2 subsets (53, 54), relies on the action of a lineage-specific transcription factor, identified as the orphan nuclear receptor RORγt (55). RORγt, encoded by RORC2, is an isoform of RORγ that belongs to the NR1F subfamily of orphan receptors, including RORα and RORβ. Previous studies have indicated that RORγt is both necessary and sufficient for Th17 cell differentiation in mouse and human CD4^+^ T cells. Ivanov et al. reported that T cells lacking RORγt (Rorc^−/−^) failed to differentiate into Th17 cells even under Th17-polarizing culture conditions, while over-expression of Rorc in naïve CD4^+^ T cells was sufficient to accelerate the expression of Th17-related cytokines and chemokines, including IL-17A, IL-17F, IL-22, IL-26, CCR6, and CCL20. Moreover, mice lacking RORγt were much less susceptible to experimental autoimmune encephalomyelitis (EAE), and CD4^+^ splenocytes from those mice could not induce the disease (55). A similarly crucial role for RORγt in human Th17 cells was also demonstrated (56). IL-6 and IL-23 signals strongly phosphorylated and dimerized signal transducer and activator of transcription 3 (STAT3), resulting in enhanced expression and nuclear translocation of RORγt, which then promoted Th17 responses by activating Th17 gene promoters, including Il17a, Il17f, Il22, Il26, Il23r, Csf-2, Ccr6, and Ccl20. In addition, IL-23 signaling-induced transcription factor Blimp-1 enhanced pathogenic Th17 function by co-localizes RORγt and STAT-3 at *Il17a, Il23r*, and *Csf-2* enhancer sites (34, 57, 58) (Figure 2). Interestingly, neither IL-23 nor IL-6 alone was sufficient to effectively generate Th17 cells (59). Nevertheless, either IL-23 or IL-6 induced IL-17 production by naïve precursors in the presence of IL-1β rather than TGF-β. T-bet + RORγt + Th17 cells were generated without TGF-β and were pathogenic in an EAE animal model, indicating an alternative pathway for Th17 differentiation (59).

Taken together, previous studies have confirmed an essential role of RORγt in the differentiation of pathogenic Th17 cells. Given that pathogenic Th17 cells play such a pivotal role in the pathogenesis of psoriasis, targeting Th17 cells, especially *via* blocking RORγt, may be a good option for treating psoriasis. In addition, RORγt might be a uniquely tractable drug target by virtue of being a nuclear receptor. Therefore, RORγt can be an attractive pharmacologic target for the treatment of Th17-mediated autoimmune diseases, including psoriasis.

## Small Molecules Targeting RORγt

Retinoid-related orphan receptor γt contains identical DNA-binding domain and ligand-binding domain (LBD). Like other nuclear receptors, the binding of ligands to the region LBD causes a conformational change, which results in recruiting transcriptional co-activators as well as activating transcriptional activity.

Since RORγt receptor was initially identified as an orphan receptor, its endogenous ligands attracted more attention at first. Previous studies have revealed that several oxysterols are endogenous modulators of RORγt activity with high-affinity. For example, 7-oxygenated sterols function as high-affinity ligands for RORγt *via* directly binding their LBDs, modulating co-activator binding, and suppressing the transcriptional activity of the receptors (60). In addition, 24S-hydroxycholestrol acts as an inverse agonist that suppresses the transcriptional activity of RORγt (61).

To develop potent synthetic RORγt ligands that selectively suppress pathogenic effector functions of Th17 cells, researchers have used many strategies to screen for potentially regulatory drug candidates, as described below.

Digoxin, the cardiotonic glycoside extracted mainly from *Digitalis lanata*, has been identified as a specific inhibitor of RORγ transcriptional activity without affecting other nuclear hormone receptors, including human androgen receptor (AR) and liver X receptor α (62). It specifically inhibits IL-17 production by Th17 cells. Moreover, it is effective in attenuating EAE in mice and decreasing the disease severity in a rat model of arthritis (62–64). However, it is toxic for human cells at high doses and may cause some adverse reactions, including arrhythmia, nausea, vomiting, blurred vision, diarrhea, depression, and even lethargy. Further studies have shown that derivatives of digoxin, such as Dig(dhd) 20,22-dihydrodigoxin-21,23-diol, and Dig(sal) digoxin-21-salicylidene, specifically inhibit the differentiation of Th17 cells in human CD4^+^ T cells without significant toxicity (62), indicating that nontoxic derivatives of digoxin may be utilized as chemical templates for the development of RORγt negative regulators.

SR1001, a derivative of liver X receptor agonist, is capable of suppressing the transcriptional activity of RORα and RORγ (65). It is a high-affinity synthetic ligand that can bind the LBD of RORα and RORγ, resulting in inhibition of murine Th17 cell differentiation and IL-17 expression by inducing conformational changes that in turn suppress the receptors’ transcriptional activity. Thence, SR1001 might be an attractive lead compound for drug development to treat Th17-mediated autoimmune diseases, such as psoriasis as well as RORα- and RORγ-mediated metabolic diseases (66, 67).

SR2211, a derivative of SR1001, only binds the LBD of RORγ and inhibits the transcriptional activity of RORγ without affecting RORα function (68). In addition, SR2211 suppresses the intracellular expression of IL-17 and has potential utility for the treatment of inflammatory diseases, such as experimental arthritis (69, 70). SR2211 has been shown to diminish genome-wide AR binding, H3K27ac abundance and expression of the AR target gene networks, and it could serve as a potential drug for the treatment of castration-resistant prostate cancer (71).

Ursolic acid (UA), a small molecule present in medicinal herbs such as *Prunella vulgaris* L., effectively inhibits the function of RORγt, resulting in greatly reduced IL-17 expression in both developing and differentiated Th17 cells (72, 73). However, UA also has other cellular targets, including the liver kinase B1–AMP-activated protein kinase (74), the NFE2-related factor 2 (75), nuclear factor-κB (76), and STAT3 pathway (77, 78), suggesting that it is not RORγt-specific *in vivo*.

TMP920, which can displace RORγt from its target loci, suppresses Th17 cell differentiation and Th17 signature gene expression (79). Based on TMP920, additional inverse agonists are developed, including TMP778, which exhibits an increase in potency and specificity. It predominantly affects RORγt transcription without removing DNA binding (79). Interestingly, the diastereomer of TMP778 or TMP776 displays no inverse agonist activity against RORγt. In experiments *in vivo*, TMP778 suppresses imiquimod-induced cutaneous inflammation and attenuates EAE. Furthermore, TMP778 also reduces expression of Th17-signature genes in cells isolated from the blood and skin of psoriatic patients (80).

Other RORγt inverse agonists have also been discovered. Using a scaffold hybridization strategy, a series of carbazole carboxamides are found to be potent RORγt inverse agonists (81). In addition, MG 2778, a cyclopenta[a]phenanthrene derivative, is identified as a lead compound for developing synthetic steroidal inverse agonists of RORγt (82). Furthermore, TAK-828F, a potent and selective RORγt inverse agonist, strongly inhibits Tc17 and Th17 cell differentiation from naive T cells and memory CD4^+^ T cells without affecting Th1 cell differentiation (83). In another study, Barbay et al. have identified 6-substituted quinolines as modulators of RORγt using a RORγt-driven cell-based reporter assay. They have further elucidated the interaction between 6-substituted quinolones and RORγt in an X-ray crystal structure (84). Moreover, A213, a potent and selective antagonist of RORγt, is found to inhibit Th17 cell differentiation *in vitro*. It also attenuates psoriatic skin lesion in two different mouse models by suppressing IL-17 production (85).

Taken together, previous studies have implicated a potential therapeutic application of RORγt antagonist for the treatment of Th17-mediated diseases, including psoriasis. Especially, targeting RORγt for the treatment of cutaneous inflammatory disorders may afford additional therapeutic benefits over existing modalities, in which only one Th17 cytokine such as IL-17A is targeted. However, the small molecules targeting RORγt could generate unwanted or unexpected results given that they may exert off-target effects *in vivo*. Those molecules must undergo rigorous clinical trials prior to a clinical application to carefully evaluate their potential side effects. In addition, other types of immune cells, including type 3 innate lymphoid cells, CD8^+^ IL-17-producing (Tc17) cells, γδT, and even Treg cells, may also express RORγt. Target RORγt could affect these cells as well. Thus, strategies targeting RORγt in Th17 cells are preferred so that we can attenuate Th17-mediated inflammation while limiting potential side effects.

## Summary and Outlook

Since there are many limitations of traditional and biological treatments for psoriasis, it is important to develop more effective and safer therapies of psoriasis. The finding of RORγt/Th17/IL-17 signaling pathway has provided further insights into the pathogenesis of psoriasis. Compared with antibodies targeting IL-17A or IL-17R alone, targeting Th17 cells themselves might benefit psoriatic patients to a greatest extent by impacting multiple proinflammatory cytokines (IL-17A, IL-17F, IL-22, and GM-CSF) that are likely to act synergistically to drive psoriatic inflammation. Hence, targeting RORγt *via* small molecule inverse agonists is a promising strategy for treating psoriasis *via* suppressing Th17 cell differentiation. Furthermore, small molecules disrupting RORγt are also expected to be safer than global immunosuppressive agents, such as cyclosporine. However, there are several challenges that need to be overcome. Researchers should generate safer and more potent compounds. Moreover, rigorous clinical studies are needed to assess their actual clinical efficacy and side effects since they could generate off-target effects. In conclusion, given the importance of Th17 cells and their proinflammatory cytokines in the pathogenesis of psoriasis, targeting RORγt seems to be a promising approach to treating psoriasis effectively and perhaps safely.

## Ethics Statement

The epidemiological data were cited without any commercial or financial uses.

## Author Contributions

LT and XY wrote the manuscript; YL and HX searched the literature; ZD and GZ edited the paper.

## Conflict of Interest Statement

The authors declare that the research was conducted in the absence of any commercial or financial relationships that could be construed as a potential conflict of interest.
